# Host Genetics and Environmental Factors Regulate Ecological Succession of the Mouse Colon Tissue-Associated Microbiota

**DOI:** 10.1371/journal.pone.0030273

**Published:** 2012-01-17

**Authors:** Philip Smith, Jay Siddharth, Ruth Pearson, Nicholas Holway, Mark Shaxted, Matt Butler, Natalie Clark, Joanna Jamontt, Robert P. Watson, Devika Sanmugalingam, Scott J. Parkinson

**Affiliations:** 1 Novartis Institutes for Biomedical Research, Gastrointestinal Disease Area, Horsham, West Sussex, United Kingdom; 2 Novartis Institutes for Biomedical Research Information Technology, Horsham, West Sussex, United Kingdom; 3 Novartis Institutes for Biomedical Research, Developmental and Molecular Pathways, Basel, Switzerland; Institute for Genome Sciences, University of Maryland School of Medicine, United States of America

## Abstract

**Background:**

The integration of host genetics, environmental triggers and the microbiota is a recognised factor in the pathogenesis of barrier function diseases such as IBD. In order to determine how these factors interact to regulate the host immune response and ecological succession of the colon tissue-associated microbiota, we investigated the temporal interaction between the microbiota and the host following disruption of the colonic epithelial barrier.

**Methodology/Principal Findings:**

Oral administration of DSS was applied as a mechanistic model of environmental damage of the colon and the resulting inflammation characterized for various parameters over time in WT and Nod2 KO mice.

**Results:**

In WT mice, DSS damage exposed the host to the commensal flora and led to a migration of the tissue-associated bacteria from the epithelium to mucosal and submucosal layers correlating with changes in proinflammatory cytokine profiles and a progressive transition from acute to chronic inflammation of the colon. Tissue-associated bacteria levels peaked at day 21 post-DSS and declined thereafter, correlating with recruitment of innate immune cells and development of the adaptive immune response. Histological parameters, immune cell infiltration and cytokine biomarkers of inflammation were indistinguishable between Nod2 and WT littermates following DSS, however, Nod2 KO mice demonstrated significantly higher tissue-associated bacterial levels in the colon. DSS damage and Nod2 genotype independently regulated the community structure of the colon microbiota.

**Conclusions/Significance:**

The results of these experiments demonstrate the integration of environmental and genetic factors in the ecological succession of the commensal flora in mammalian tissue. The association of Nod2 genotype (and other host polymorphisms) and environmental factors likely combine to influence the ecological succession of the tissue-associated microflora accounting in part for their association with the pathogenesis of inflammatory bowel diseases.

## Introduction

Inflammatory bowel disease (IBD), encompassing Crohn's disease and ulcerative colitis, is characterized by chronic and relapsing inflammation of the gastrointestinal tract. The exact cause of this spectrum of diseases is unknown, although it is likely that the characteristic chronic, relapsing and remitting inflammation is derived from a complex interplay of environmental (such as diet, smoking and stress) and genetic factors leading to a robust host immune response to the intestinal microbiota [1]–[9].

Various preclinical *in vivo* models also implicate bacteria as causative agents of gastrointestinal inflammation - germfree status prevents development of gut and joint inflammatory disease and anatomic location of inflammation is bacterial strain-dependent [10], [11]. There are a number of genetic mouse models that have attempted to reconstruct a disease-relevant intestinal inflammatory phenotype. With a few exceptions, attempts to generate models with comparable phenotypes to the human condition have not been successful or limited in the spectrum of disease location and characteristics. While this has frustrated efforts to identify the molecular mechanisms underlying chronic inflammatory disease of the intestine, lack of a disease-like phenotype in these models also provides an opportunity to investigate fundamental questions regarding the pathogenesis and development of chronic disease.

Nod2 was originally identified as a Crohn's disease susceptibility factor by linkage analysis that was subsequently confirmed in genome-wide association studies [12], [13]. Efforts to reproduce a Crohn's-like phenotype in several Nod2 mouse models have been unsuccessful but did highlight various roles for Nod2 in immune response, development and microbial defence [14]–[16]. In particular Nod2 deletion did not correlate with an enhanced inflammatory response in the DSS-damage model, but had a clear role in microbial defence against a spectrum of bacteria. These infection model studies highlight an important role for Nod2 in bacterial pathogen defence, however, in the context of inflammatory bowel disease the commensal microbiota is likely the underlying cause of inflammation.

The role of bacteria in the commonly used acute model of DSS-induced colitis is complex. In germ-free mice mucosal injury is exacerbated demonstrating the protective effect of bacterial induced NF-κB signalling in epithelial cell homeostasis [17], [18]. However, in conventionally housed mice antibiotic treatment ameliorates DSS –induced colitis, suggesting that bacteria drive inflammation and epithelial damage [19]. The DSS damage model, therefore, presents itself as a mechanistic model to investigate the integration of host genetic polymorphisms in pattern recognition receptors, like Nod2, and environmental damage that exposes the host to the commensal flora of the colon.

In this study we used DSS damage to characterise the time course of bacterial infiltration of the colon tissue, to examine the immune response of the host in response to this infiltration, and to determine the influence of Nod2-deletion on the ecological succession of the tissue-associated microbiota. The results presented demonstrate that environmental damage and genetics integrate to regulate ecological succession of the microbiota – a concept that likely plays a role in the pathogenesis of chronic inflammatory diseases of the intestine.

## Results

Dextran sodium sulfate (DSS) is a commonly used agent to investigate the molecular mechanisms mediating inflammation of the gastrointestinal tract and the efficacy of potential therapeutic agents in rodents *in vivo*. In previous reports, Melgar and colleagues demonstrated that a single DSS exposure of C57Bl/6 mice for 5 days was sufficient to induce an acute colitis that progressed to severe chronic inflammation [20], [21]. Administered in the drinking water, DSS leads to development of acute inflammation of the mucosa several days after administration, perhaps as a result of bacterial penetration of the sterile mucous layer of the colon [22]. We used DSS damage as a mechanistic model of environmental factors that result in disrupted epithelial barrier function to investigate the regulation of host/commensal interactions *in vivo*.

### Phenotypic development of intestinal inflammation following DSS damage

**Figure 1:**
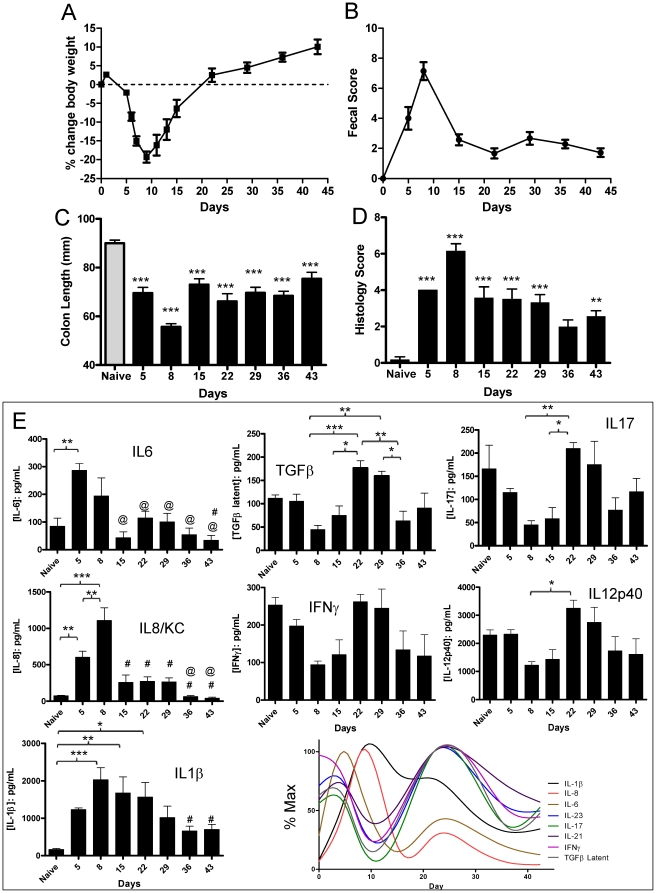
Temporal physical, histological and biomarker analysis of DSS damage. Mice were administered DSS in their drinking water from day 0 to day 5 and individual parameters outlined in each panel assessed by criteria described previously [41]. * = p≤0.05, ** = p≤0.01, *** = p≤0.001 by 1-way Anova with Bonferroni's multiple comparison test. **A.** Body weight (physiological parameter for progression of inflammation) expressed as mean +/− SEM, n = 7. **B.** Fecal Score (combined weighted score of intestinal damage based on the presence of blood in the stool and subjective assessment of diarrhea) as a physiological indicator of colon tissue damage expressed as mean +/− SEM, n = 7. **C.** Colon length (physiological parameter for progression of inflammation) expressed as mean +/− SEM, n = 7. Statistical comparisons are versus Naive (no DSS-treatment) mice. **D.** Histology Score (quantitative assessment of microscopic tissue damage identical to that described previously [41]) expressed as mean +/− SEM, n = 7. Statistical comparisons are versus Naive (no DSS-treatment) mice. **E.** Temporal cytokine (as indicated) profiles in colon homogenates from treated mice expressed as mean +/− SEM, n = 7. Statistical comparisons by Anova/Bonferroni. @ = p≤0.05 vs day 5, # = p≤0.05 vs day 8.

Mice were treated with DSS in the drinking water *ad libitum* for 5 days, and groups sacrificed over time to assess the environmental damage (Figure 1). As expected, DSS administration on days 1 to 5 resulted in a significant 20% decrease in body weight by day 8 (Figure 1A). The mice slowly regained weight until the end of the experiment on day 43. Weight gain was mirrored by significant temporal changes in fecal score, colon length, and histology (Figure 1B, C, and D). None of these three parameters returned to untreated levels and remained significantly elevated relative to controls consistent with development of a chronic inflammatory state in the mice previously observed [20], [21]. Cytokine biomarkers from tissue homogenates were examined to characterize the inflammatory status of the mice (Figure 1E). These data generally confirmed the clinical parameters although there were some interesting temporal changes reflecting the character of the developing pathology. The time course of IL1β protein mirrored the observed changes in the clinical parameters peaking at day 8 and subsequently decreasing to the end of the experiment. Similar to IL1β, IL6 and KC/IL8 also peaked on days 5 and 8 respectively, and returned to baseline levels. IL12p40, IL17, TGFβ and IFNγ demonstrated parallel temporal profiles over the course of the experiment. All four cytokines did not differ from control levels during the initial inflammatory response but temporally peaked at day 22; for TGFβ, IL12p40 and IFNγ levels were significantly higher than cytokine levels on day 8. Overall, the results of the clinical, histological and inflammatory biomarkers examined support the conclusion that DSS-induced epithelial damage results in development of a maturing immune response from an initial acute phase into a chronic inflammation of the colon characterized by distinct proinflammatory cytokine profiles.

### Temporal regulation of bacterial/host interaction following DSS damage

**Figure 2:**
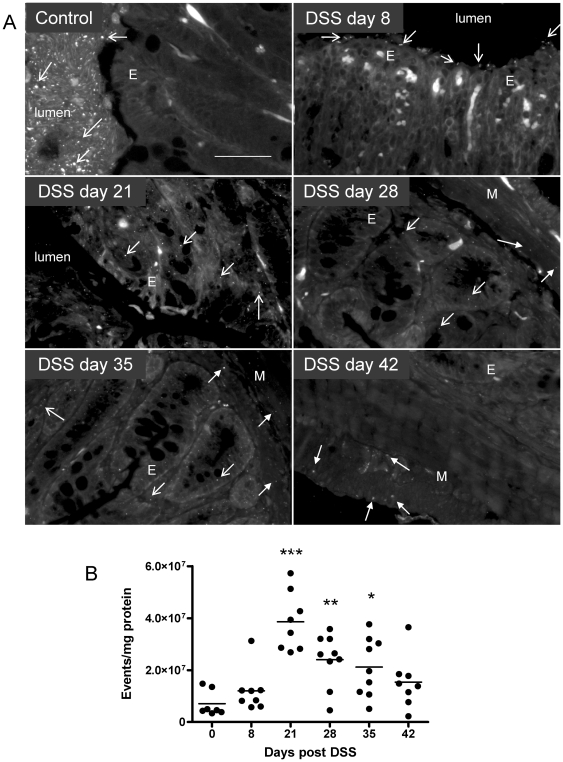
Proximity and progression of host/commensal interactions following DSS damage. **A.** Localisation of commensal bacteria by EUB338 FISH probe analysis of FFPE sections from DSS-treated mice. Open arrows highlight examples of luminal or mucosal bacteria, closed arrows indicate bacteria associated with the muscle layers. E, epithelium. M, muscle. Bar = 50 µm. **B.** Quantification of colon tissue bacteria load in individual mice by FACS following DSS damage. * = p≤0.05, ** = p≤0.01, *** = p≤0.001 vs day 0 (no DSS treatment) by 1-way Anova with Bonferroni's correction.

A previous report demonstrated that DSS administration is associated with penetration of the colonic tissue by the commensal flora of the intestine [22]. Therefore, we tested the hypothesis that bacterial penetration of the tissue regulates the progression to chronic inflammation (Figure 2). A cocktail of fluorescent-labeled bacterial FISH probes recognizing >95% of type strain bacteria was used to gauge bacterial infiltration into the tissue following DSS damage (Figure 2A, Figure S1). In the absence of DSS damage, the commensal bacteria remained in the lumen of the gut separated from the host by the mucous layer (Figure 2A). Following DSS, a clear progression of commensal infection into the host tissue could be observed. Consistent with the previous report, at day 8 following DSS damage, commensal bacteria were reliably observed in association with the epithelial layer. Tissue-associated bacteria were detected in deeper tissue layers over the time course of the experiment. By day 21, commensal bacteria could be detected in the lamina propria of the mucosa and by day 28 in the submucosal and muscle layers. From day 35 to day 42, complete histological restitution of the epithelial layer was observed. However, there remained clear evidence of persistent commensal bacteria infiltration of deeper tissue layers especially the muscle and viscera. The location of the tissue-associated bacteria, therefore, correlated with the observed changes in the inflammatory cytokine profiles. The persistent infiltration of the colon with commensal bacteria likely contributes to the chronic inflammatory state following DSS damage of the epithelium in C57BL/6 mice.

We examined the tissue loads of commensal bacteria over the time course of the *in vivo* experiment. A significant proportion of the gastrointestinal microbiota is not cultureable making an accurate quantitative assessment of undefined bacteria difficult. We circumvented this problem by adapting two previously defined methods to detect bacteria from intestinal samples [23], [24]. The method was validated in a series of experiments summarized in Figure S2. Pieces of the colon were removed and treated with gentamicin – a non-cell permeable antibiotic to reduce bacteria associated with the tissue that had not penetrated inside the cells of the host. In the acute phase of colonic inflammation up to day 8, no significant increase in tissue-associated bacterial numbers was observed (Figure 2B). By day 21 however, tissue-associated bacterial counts were significantly increased. These numbers remained significantly elevated up to day 35. Together, the data presented in Figure 2 demonstrates that DSS damage triggers a progressive infiltration of the host with the commensal flora from the colonic lumen. The bacteria invade deeper tissue layers over time eventually reaching and colonizing the submucosa and muscle layers of the gastrointestinal tract.

### Immune cell infiltration following DSS damage and correlation with bacterial penetration

**Figure 3:**
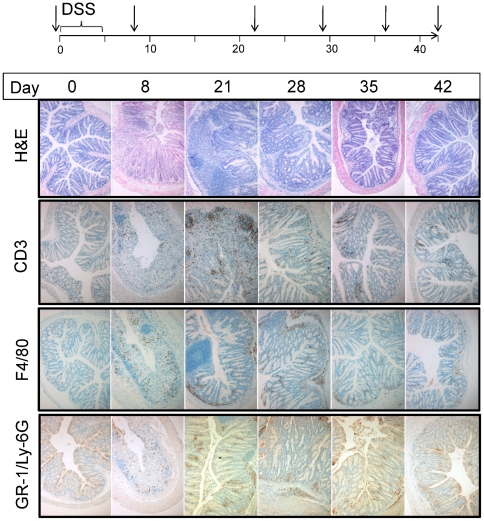
Infiltration of colon by immune cells assessed by immunohistochemistry following DSS damage. Top panel provides timeline for the experiment with arrows indicating times of histological and immunohistochemical assessment. Representative images for each of the cell markers and from each of the time points are shown. H&E (haematoxylin/eosin histochemical stain), CD3 (T cell marker), F4/80 (macrophage marker) and GR-1/Ly-6G (granulocyte marker).

Since the cytokine profiles demonstrated temporal regulation correlating with the compartmentalization of the tissue-associated bacteria, we examined the infiltration of specific immune cells into the colon over the time course of the experiment to determine how the residence of commensal bacteria in the colonic tissue correlates with the development of the immune response of the host. We examined levels of selected immune cells in the tissue across the time course of the experiment by IHC (Figure 3). As demonstrated by CD3, F4/80 and GR-1/Ly-6G positive staining, a significant infiltration of the colonic mucosa by T cells, macrophages and granulocytes was observed by day 8, post DSS. These cell types accumulated in the tissue up to day 22–29. While the markers were apparent in the mucosa on day 8, by day 22 the F4/80 macrophage staining partitioned primarily into the sub-mucosal layer adjacent to the muscle while T cells and granulocytes were recruited to the damaged mucosa and lamina propria. All cell types were visible in the tissue until day 42 of the experiment. The data outlined above demonstrate a correlation between the DSS-dependent histological damage and cytokine profiles observed in Figure 1, with the tissue penetration of the commensal flora observed in Figure 2 and recruitment of immune-related cells into the colon in Figure 3. The data described are consistent with a model where the loss of bacterial compartmentalization initiates an immediate immune response from days 5 to 15 as demonstrated by elevated epithelial-derived chemokines and cytokines as well as recruitment of CD3+ T cells, F4/80 macrophages and GR-1/Ly-6g positive granulocytes into the colon. The accumulation and location of bacteria in the tissue correlates with the peak in immune cell infiltration into the tissue on day 21 and subsequent reduction in bacterial numbers.

### Interdependence of bacterial infiltration and host immune response

The correlation of bacterial infiltration and immune cell recruitment into the colon led us to examine the causal relationships between the bacteria and the host immune response in the chronic inflammatory phase. We treated mice with DSS and allowed them to recover to the approximate end of the acute inflammatory phase on day 15. At this time, mice were treated with either dexamethasone to suppress inflammation or with an antibiotic cocktail targeting the bacteria followed by assessment of histological and clinical parameters on day 22 (Figure S3). Both treatments significantly improved the condition of the mice. The gross pathological readouts of inflammation like colon length as well as the associated histological scores significantly improved with either treatment. As expected, antibiotic treatment reduced detection of tissue-associated bacteria. Interestingly, inhibition of the immune response with dexamethasone also significantly decreased tissue-associated bacterial levels. These data demonstrate interdependence of the tissue-associated bacteria and chronic inflammation observed in the mice day 21 post-DSS - the chronic inflammatory state supports the residence of the commensal flora within the tissue and the resident bacteria play a significant role in the pathologic consequences of inflammation within the host.

### Nod2 regulates tissue-associated bacterial loads independent of host immunity

Having established a clear correlation between environmental damage of the epithelium, host inflammation and the interaction between the commensal flora and the host, we turned our attention to host genetic factors regulating host/commensal interactions. Pattern recognition receptors (PRR) play an important role in the detection of foreign organisms that penetrate host defences and likely play an important role in the recognition and clearance of the tissue-associated bacteria observed in this model.

**Figure 4:**
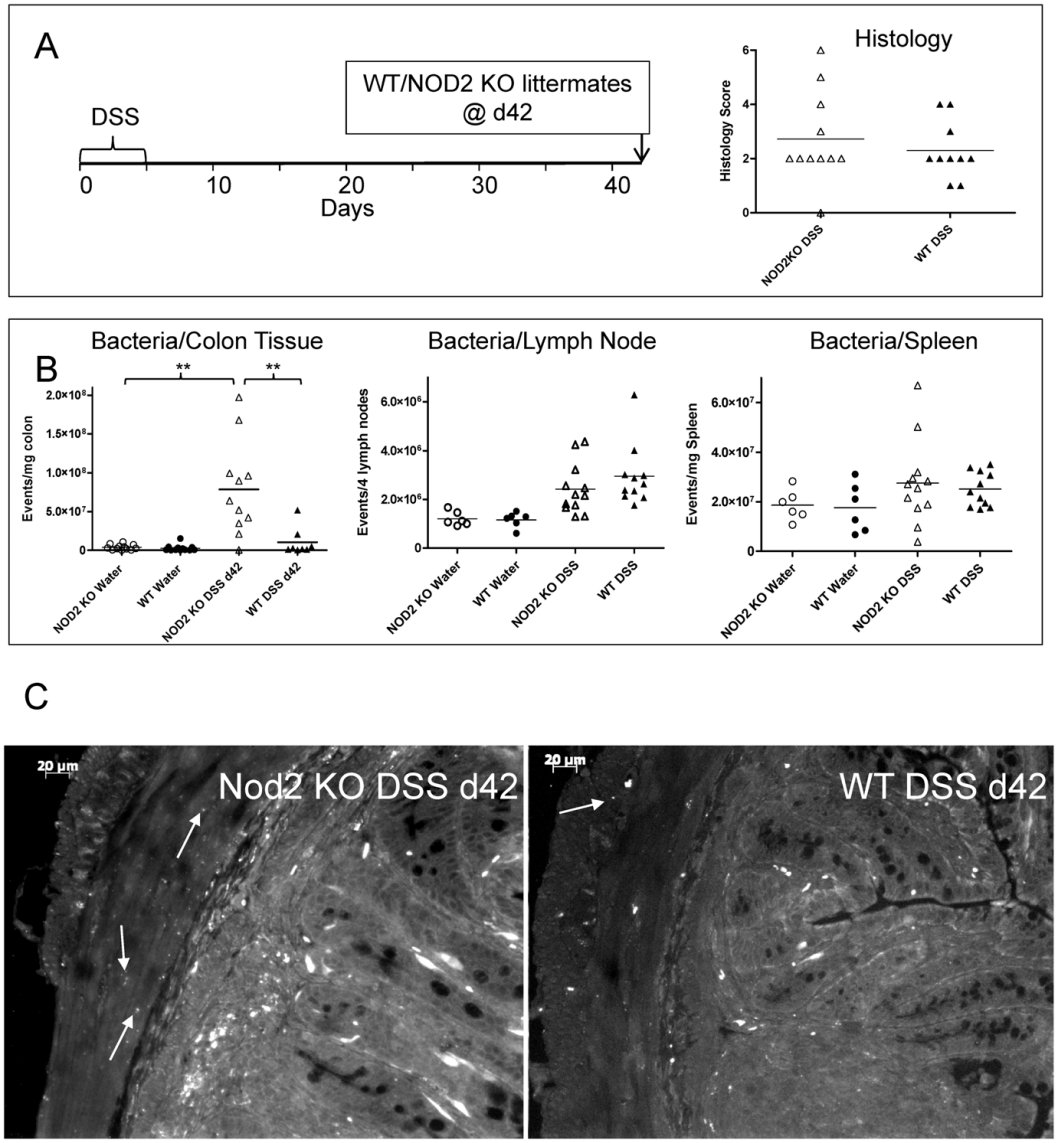
Comparison of physical and histological parameters and bacterial load of WT and Nod2 KO littermates following DSS damage. **A.** Timelines and histology assessment for individual mice. No significant difference was observed between the two genotypes for physical parameters (body weight loss, colon length: not shown) nor histological scores between the two groups (see Figure S4 for data from 2 additional independent experiments). **B.** Colon, mesenteric lymph node and spleen tissue-associated bacterial loads assessed by FACS 42 days following DSS damage. ** = p≤0.01 by Anova with Bonferroni's multiple comparison test. **C.** Residence of commensal bacteria in the muscle layer in Nod2 KO mice. Examples of bacterial staining by EUB338 FISH probes are indicated by closed arrows in these representative images.

Nod2 is a cytoplasmic PRR that is genetically-associated with Crohn's disease and has been demonstrated to play an important role in pathogen defence in mouse models [25], [26]. Previous reports have demonstrated that deletion of Nod2 has no impact on the severity of DSS-induced colitis in mice; observations that we reproduced (not shown) [14]. These published observations were made 9 days after initiation of the experiment correlating with the acute phase of inflammation we have characterized in the current study. Therefore, we investigated the role of Nod2 at time points associated with the developing pathology of disease in the DSS model (Figure 4A). Firstly, we examined the bacterial loads in the colonic tissue of mice at the day 9 (acute inflammation), day 21 (beginning of chronic inflammation) and day 42 (chronic inflammation) time points. No significant differences in the numbers of tissue-associated bacteria were observed in naive WT vs Nod2 KO mice or mice examined on day 9 and 21 following DSS treatment (data not shown). On day 42, however, a significant increase in the bacterial load was observed in mice that were Nod2 deficient (Figure 4B). In Figure 2, DSS damage led to infiltration of deeper tissue layers with commensal bacteria over time. This progression was also observed in the Nod2 KO mice when compared with WT littermates on days 8, 24 and 42 post-DSS demonstrating that the elevated bacterial levels observed by quantitative FACS analysis on day 42 primarily reside in the sub-mucosal and muscle layers (Figure 4C). This elevated bacterial load occurred without significant or proportional increases in host inflammation as demonstrated by gross morphological and histological readouts for DSS treated WT and Nod2KO mice on day 42 post-DSS (Figure 4A, Figure S4). Despite significantly elevated bacterial loads in the Nod2 KO mice, no significant difference in colon tissue-associated cytokines or serum antibody levels (IgA, IgG1, IgG2a, IgM, IgE) could be detected between WT and Nod2 KO littermates (Figure S4, S5, S6).

### Nod2 regulates bacterial levels in the colon but not secondary lymphoid organs

Previous reports have demonstrated that Nod2 mediates mucosal, but not systemic defence against *Listeria* infection as demonstrated by significant susceptibility of Nod2 KO mice to infection by bacteria delivered by the oral, but not intraperitoneal or intravenous routes [14]. Therefore, we also examined bacterial loads in secondary lymphoid organs on day 42 post-DSS (Figure 4B). Complementing the previous observations with *Listeria*, Nod2 status did not correlate with any significant changes in the bacterial load of peripheral lymph nodes or the spleen suggesting that Nod2 plays an important role in local bacterial clearance of the colon contributing to the barrier function of the gastrointestinal tract.

### Nod2 does not regulate richness nor diversity of colon tissue-associated bacterial community

We have previously shown that purified Nod2 LRR domains directly interact with a broad range of bacteria in culture and demonstrate direct antibiotic activity [27]. Since elevated bacterial loads were observed in Nod2 KO mice in the absence of any significant increase in histological or biomarker-related readouts of inflammation that could contribute to changes in the tissue-associated bacterial community structure, we explored the composition of the tissue-associated flora to account for the Nod2-dependent deficiency in bacterial defence. Four colon-derived libraries (WT H_2_O, Nod2 KO H_2_O, WT DSS, and Nod2 KO DSS) of full length 16S rRNA sequences were generated from groups of WT and Nod2 KO mice sacrificed on day 42, when a significant increase in tissue-associated bacteria were observed in Nod2 KOs following DSS damage (Figure S7).

### Nod2 and environmental damage independently regulate the bacterial community structure

**Figure 5:**
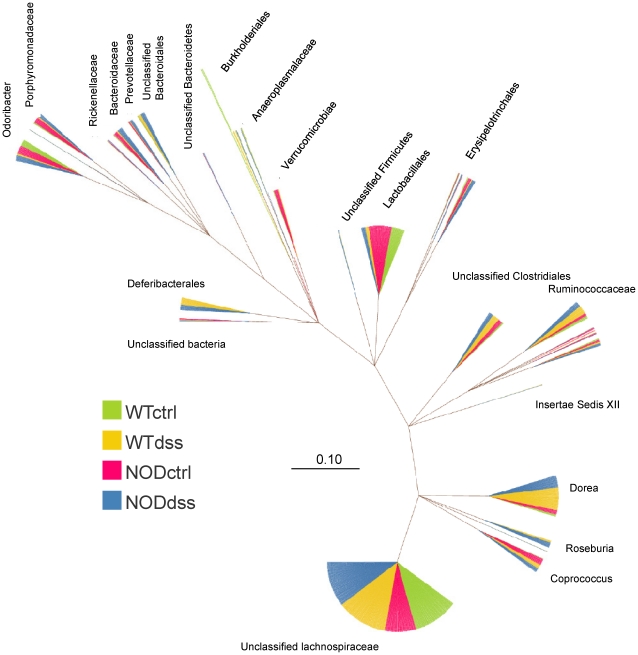
Phylogenetic tree visualisation of full length 16S rRNA sequences from WT and Nod2 KO mouse colon tissue 42 days post DSS or control as indicated. See Materials and Methods for parameters.

We examined the impact of genotype and/or environmental DSS damage on the community structure in each of the groups. Analysis of the phylogenetic tree (Figure 5) generated from these experiments indicated that the dominant ‘leaves’ identified included clusters in the ‘unclassified lachnospiraceae’, dorea, and lactobacillales bacteria. The lachnospiraceae cluster was evenly distributed between the 4 groups. However, the dorea cluster was composed predominantly by the DSS-treated groups while the lactobacillales cluster was primarily derived from the non-treated control mice groups indicating unique changes in the composition of the community structure between the treatment groups. Other minor clusters demonstrated specificity for specific genotypes. For example, the bacteroidaceae cluster was dominated by Nod2-derived sequences. Overall, observation of the clusters within the phylogenetic tree indicated that both genotype and DSS damage influenced the community structure of the bacteria identified.

**Figure 6:**
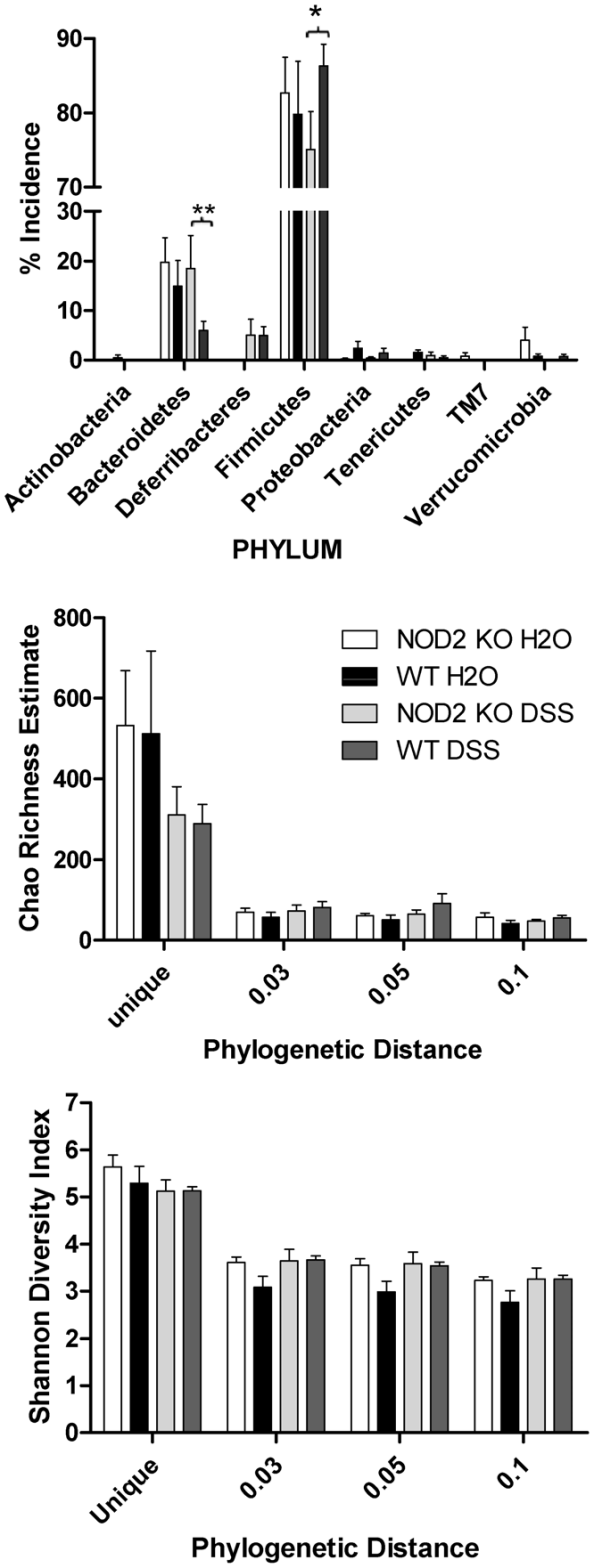
Richness, diversity and taxonomic analysis of the WT and Nod2 KO colon tissue-associated bacterial communities. Top panel: Incidence of phyla from treatment groups of WT and Nod2 KO littermates. Mean +/− SEM (n = 4–6) for each group is shown. * p≤0.05, ** p<0.01 by 2 way ANOVA with Bonferroni's multiple comparison test. Bottom panels: the Chao and Shannon estimates for richness and diversity were calculated from individual mice from each group as indicated (n = 4–6). The mean +/− SEM are shown. No differences were statistically significant by ANOVA.

We attempted to determine the taxonomic classification of the populations associated with each of the groups using the RDP database and BLAST-based alignments of the 1700 full length 16S rRNA sequences. We classified the sequences from the phylum to genus level based on the ‘nearest hit’ to the input sequence found in RDP (Figure 6, Figure S9). At the genus level, less than 40% of the sequences were assigned a classification with >95% confidence score and therefore we focused our statistical analysis on those sequences assigned genus classification greater than this threshold (Figure S8). Using these parameters, no significant changes in genus frequency could be observed between WT and NOD2 KO littermates in the presence or absence of DSS treatment (2-way ANOVA with Bonferroni's correction).

Previous reports demonstrated a significant decrease in firmicutes and significant increase in bacteroidetes associated with the Nod2 KO genotype in ileal tissue [28]. We examined the classification at the phyla level of the colon-associated microbiota using the same ‘nearest hit’ approach as described for the genus classification, above (Figure 6, Figure S9). Confidence in the phyla classification was significantly higher than that at genus level – more than 99.9% of the sequences were classified with >99% confidence (not shown). Firmicutes and bacteroidetes were the dominant phyla observed in all the samples consistent with previous reports. The individual samples (n = 4–6 for each of the groups) associated with each of the genotypes and treatments were examined to statistically evaluate any significant differences between the four groups. This analysis demonstrated that the elevated tissue-associated bacteria 42 days after DSS treatment in the Nod2 KO mice also correlated with a significant increase in the prevalence of the bacterioidetes and significant decrease in the firmicutes phyla in Nod2 KO mice relative to WT littermates (Figure 6). This difference, consistent with the previous report using ileal tissue, was only observed following DSS damage and not in control animals. This data, in conjunction with the increase in bacterial infiltration demonstrated in Figure 4, supports the conclusion that Nod2 deficiency is permissive for some bacteroidetes bacteria to establish a niche in the colon.

We tested the hypothesis that host genetics and environmental damage have a significant effect on the community structure of the colon tissue-associated bacterial flora using phylogenetic distance-based statistical methods independent of classification. The richness and diversity of the colon-associated community structures of the 4 groups was assessed by the Chao Richness Estimate and Shannon Diversity Index (Figure 6). No significant differences could be observed between any of the groups, suggesting that neither Nod2 genotype nor the environmental impact of DSS damage made a substantial contribution to the overall biodiversity in the mice. Sequences from the four groups were compared using Libshuff and Parsimony statistical tests for comparison of communities (Table 1). Using both statistical tests and in all cases, each group of sequences was distinct from the others. From the data presented regarding the microbial communities, we conclude that host Nod2 genotype and DSS damage independently and significantly impact the structure but not the diversity nor the richness of the colon tissue-associated microbial community.

**Table 1:** Community Structure Comparison: Libshuff and Parsimony Statistical Analysis of Colon-Associated Bacterial Populations.

	Libshuff			*Parsimony*	
Comparison	dCXYScore	P	Significance	*Pars Score*	*Significance*
NOD2KOctrl-NOD2dss	0.00155752	<0.0001	p<0.0001	146	<0.0010
NOD2KOdss-NOD2KOctrl	0.00549762	<0.0001			
NOD2KOctrl-WTctrl	0.00253056	<0.0001	p<0.0001	125	<0.0010
WTctrl-NOD2KOctrl	0.00140359	<0.0001			
NOD2KOdss-WTdss	0.00059479	<0.0001	p<0.0001	175	<0.0010
WTdss-NOD2KOdss	0.00125859	<0.0001			
WTctrl-WTdss	0.00185298	<0.0001	p<0.0001	109	<0.0010
WTdss-WTctrl	0.01029907	<0.0001			

## Discussion

A single layer of epithelial cells are responsible for development of the physical barrier that separates the host from coming into direct contact with the 10^12^ bacteria that compose the commensal flora [29]. Among other functions, these bacteria nourish their hosts, prevent infection by pathogens, influence gastrointestinal tract development and the maturation of the host immune system. On occasions when the epithelial barrier is disrupted, these essential symbiotic organisms can opportunistically escape their niche in the intestinal lumen and come into direct contact with their host where an immune response is initiated. Circumstances where the infection cannot be cleared or tolerance to the commensal flora is lost, could lead to a chronic inflammatory state. The interaction between the host and the microbiota of the gastrointestinal tract, therefore, play important roles in both health and disease.

The epithelial cells of the colon are responsible for development of the physical barrier that separates the host from coming into direct contact with the commensal flora. Mucous producing goblet cells are more abundant in the bacteria-dense colon than in the small intestine, where bacteria are present at orders of magnitude lower concentrations. There is some evidence that mucous thickness and the continuity of the mucous barrier correlates with disease severity of ulcerative colitis [30], [31]. DSS-induced colitis is often used as a model of epithelial barrier disruption to test clinical compounds and explore the molecular mechanisms underlying colonic inflammation in the preclinical setting. DSS treatment results in progressive loss of the mucous layer and direct contact of the commensal flora of the gastrointestinal tract with the host resulting in a robust inflammatory response [22]. In this study we considered DSS damage of the colonic mucous layer as a mechanistic model of environmental factors (eg. drugs, smoking, alcohol, allergens and pathogens) that can potentially disrupt the epithelial barrier of the colon bringing the commensal flora and the host in proximity to each other. The data presented demonstrate that initiating contact between the host and commensal bacteria results in a progressive development of the host immune response resulting in clearance of the bacterial load over time. Clearance of bacteria occurs with a significant impact on the community structure of the tissue-associated microflora as demonstrated by statistical examination of the composition of the commensal bacteria before and after damage (Table 1). Host genetics - as determined in our studies by the comparison of WT mice with Nod2 littermates –significantly regulates the accumulation of bacteria following DSS damage in Nod2 KO animals (Figure 4, Figure S4) playing a significant role a role in reestablishing host/bacterial homeostasis. Nod2 does not only regulate bacterial clearance; as demonstrated by our study and others its presence or absence has a significant impact on the community structure of the tissue-associated commensal community structure [28].

Taken together, the data presented in this study experimentally demonstrate the contribution of genetic and environmental factors in the ecological succession of the commensal flora. In the womb, mammals reside in a sterile environment acquiring their flora upon birth. This is influenced by the mode of delivery as demonstrated by previous publications establishing significant differences in the microbiota of infants delivered by caesarean section [32]–[34]. Our data would support the original hypothesis put forward by Ley et al. that the acquisition and subsequent homeostasis of the microbiota is regulated by the genetic makeup of the individual depending on the interaction between innate and adaptive immune mechanisms and the composition of pattern-recognition receptors, such as Nod2 [35]. After birth, individuals become exposed to diverse diets (breast fed vs formula), therapeutic interventions (antibiotics and other drugs) and pathogens (viral, bacterial and parasites) all thought to individually effect the composition of the flora [36]–[39]. As humans age, they continue to be exposed to a new range of experiences and associated environmental challenges; weaning, school, smoking, alcohol, more drugs, migration [40]. In turn, all of the events that occur to reestablish a new homeostasis of the host and flora would be regulated by the genetic makeup of the host potentially accounting for genetic susceptibility to developing chronic inflammatory diseases, like IBD. Each individual will have a unique experience in terms of the types of damage leading to exposure to their microbiota as well as the order of occurrence conferring a unique set of evolutionary pressures. In the eventuality that the evolved microflora is not tolerated, or cannot be cleared by the immune system chronic inflammation would be the result. The disease would also be influenced by the established niche. For example, in our study Nod2 did not a significant contribution to control bacterial levels until they reached submucosal layers over time. If this were the case in humans, the genetic susceptibility loci of the host could play an important role in the location of the established communities and determine transmural vs mucosal disease, disease location, and account for other variations in disease presentation. Further studies in patient samples and preclinical models will provide opportunities to test these hypotheses.

Ecological succession models have been proposed for other indications with clear association of the microbiota and host interactions, such as periodontitis. While epidemiological evidence supports the succession of tissue-associated bacteria in association with disease development, investigational studies of the underlying molecular mechanisms associated with the epidemiological data are less frequent. The goal of this study was to provide a platform for experimental evaluation of underlying principles associated with complex diseases coupled with disrupted host/commensal interactions. In this study, DSS was used as a mechanistic model of environmental damage of the epithelial layer. In the context of the gastrointestinal tract, clear associations between environmental factors, host genetics, Crohn's disease and ulcerative colitis have been demonstrated and additional experimental studies may provide an ecological explanation for their association with IBD.

## Materials and Methods

### Ethics Statement

All animal experiments were approved by the Novartis Ethical Review Process (NERP) and conducted in accordance with UK Home Office regulations (licence number PPL 70\6461).

### Reagents

Antibodies; anti-human CD3 antibody (Vector Labs, Clone Sp3, 1∶200), rat anti-mouse F4/80 (AbD Serotec, CI:A3-1, 1∶200), Rat anti-GR-1/Ly6g (Novus, MAB0866, 1∶100). FISH probes: Cy3-conjugated EUB338I, EUB338II, EUB338III (probeBase) were synthesized by MWG. Mice: Nod2 KO mice were used under licence from Yale University (Kobayashi, 2005). Detection/capture antibodies used for mouse ELISAs: biotinylated A85-1 mAb (BD553441) /anti-mouse IgG1 A85-3 mAb (BD5533445), biotinylated R19-15 mAb (BD553388)/anti-mouse IgG2a R11-89 mAb (BD553446), biotinylated C10-1 mAb (BD556978) /anti-mouse IgA C10-3 mAb (BD556969), biotinylated R35-118 mAb (BD553419), anti-mouse IgE R35-72 mAb (BD553413), biotinylated G53-238 mAb (BD553886) /anti-mouse IgM G53-238 mAb (BD553885).

### In vivo DSS model

WT and Nod2 KO littermates on C57Bl/6 background were treated with DSS in the drinking water. Histological scoring was as previously described [41].

### Bacterial quantitation by FACS

Tissue segments were removed from the distal colon, treated with 0.3 mg/ml gentamicin for 2 hours on ice, washed several times in PBS and homogenised. Homogenate was filtered using cell strainer and diluted in PBS prior to FACS analysis by forward/side scatter according to standard gate as determined for cultured bacteria (Figure S2).

### ELISAs

Serum immunoglobulin levels were assessed by capture ELISA using antibody pairs from Becton Dickinson, UK. Antibody detection was visualised using 0.1 µg/ml Streptavidin-HRP (Biosource, UK) and TMB substrate (Cambridge Bioscience, UK). The concentrations of IL1β, IL8/KC, IL6, TGFβ, IL-17, TNFα, IFNγ, and IL12p40 in homogenized colonic tissue were measured by enzyme-linked immunosorbent assay (ELISA) kits according to the manufacturer's instructions (R&D Systems).

### IHC and histochemical stains

*Avidin-biotin peroxidase immunohistochemistry* was performed on an automated Ventana Benchmark XT immunostainer (Roche) in the standard manner using 4 µm paraffin sections. For all stained sections selected high-power fields (60× magnification) were used for blinded quantification of indicated cell-associated markers by a third party. *16S rRNA FISH*: formalin fixed paraffin-embedded sections were deparaffinised, rehydrated, and fixed in 4% paraformaldehyde for 5 minutes followed by PBS washing. Tissue sections were incubated 10 minutes/RT in TE buffer containing 10 mg/mL of lysozyme and of lysostaphin prior to addition of hybridization solution (0.9 M NaCl, 20 mM Tris HCl, pH 8, 0.01% SDS, 30% formamide). Fixed tissue sections were then hybridized with 4.5 ng/µL of a 1∶1∶1 molar ratio of the EUB338I, EUB338II, and EUB338III 5′-end-Cy3-labeled 16S rRNA targeted oligonucleotides in hybridization buffer overnight at 35°C, washed in 65 mM NaCl, 20 mM Tris HCl, pH 8.0, 5 mM EDTA, and 0.01% SDS prior to mounting using Vectashield (Vector Laboratories, Burlingame, CA).

### 16S rRNA procedures and analysis (outlined in Figure S10)

#### Sample preparation

Tissue samples (0.5 cm/30 mg) were collected on ice, treated as described for FACS analysis (above) and immediately processed for DNA extraction using the Qiagen DNA easy Blood and tissue extraction kit using the protocol modification for gram negative bacterial DNA extraction as per manufacturer recommendations (yield ∼30 microgram DNA). PCR (25 cycles) was performed in triplicate (including no template controls) with varying melting point parameters (to compensate for PCR bias) using the 16S rRNA gene universal primers 8F and 1492R [42], [43]. The triplicate products were pooled and ligated into a TOPO-TA vector (Invitrogen). The resulting colonies were checked for insert by PCR and were subsequently sequenced from both ends using M13 forward and reverse primers on an ABI 3730 sequencer (minimum 120 from each sample). *Bioinformatics*. Sequences were quality checked and processed using a bioinformatics pipeline incorporated into Pipeline Pilot. The ABI trace file reads were processed using DNABaser encompassing base calling QC, primer sequence removal and contig building from forward and reverse sequences. The resulting sequences were then analysed in Mothur (1.17.3) with integrated chimera checking using a licensed Silva reference alignment as template [44]. Filtered sequences were used for assessment of microbial community structure within Mothur. Classification of the sequences was carried out within mothur using the RDP classification scheme employing the Silva database release. The classification data was then parsed and visualised using Spotfire. *Tree Building*. The sequences were aligned and formatted using myRDP and exported into ARB for tree building. The resulting tree was then labeled and visualized within ARB. The sequences used in the analysis are deposited with NCBI under the accession numbers JQ083720 to JQ085245.

## Supporting Information

Figure S1 **Bacterial FISH probe validation.** Formalin-fixed paraffin-embedded colon samples were processed and probed with EUB338 FISH probest for 16S rRNA as described in Experimental Procedures. Left panels: Cy5 probes only. Right panels: Cy5 probes in the presence of 100× excess ‘cold’ EUB338 probes to assess non-specific binding.
(TIF)

Figure S2 **Validation of FACS-based bacterial quantitation.** A. Gate selection (forward/side scatter: yellow) for bacterial quantitfication using cultured bacteria and overlap with fecal bacteria (in blue). B: Validation of quantification by comparison with EUB338 FISH probe. Both methods gave comparable statistical significance (*p≤0.05: Students T test) using samples from day 42 post-DSS Nod2 KO mouse colon. C: Validation of FACS bacterial quantification. Fecal matter from mice prior to (Pre-antibiotics) and 3 days following (Post-antibiotics) treatment with broad range antibiotic cocktail was processed and bacteria quantified by the forward/side scatter gate. (**p≤0.01: Students T test).
(TIF)

Figure S3 **Both antibiotics and steroid treatment significantly impact damage and bacterial tissue penetration.** Top left: time line for antibiotic/dexamethasone experiment. Top right: Histological score following therapeutic treatment. *p≤0.05 Students T test vs Vehicle. Bottom left: colon tissue-associated bacterial counts by FACS. *p≤0.05, ***p≤0.001 vs Vehicle group. Bottom right: Colon length (mean +/− SEM; n = 7,8) as physical parameter for tissue damage. **p≤0.01 Students T test vs Vehicle group.
(TIF)

Figure S4 **Histology and bacterial load assessment of WT and Nod2 KO littermates following DSS damage.** Histology score summary (left) and colon tissue-associated bacterial loads (right) assessed by FACS 42 days following DSS damage. * = p≤0.05 by Students T test. See Figure 4 for additional independent experimental data.
(TIF)

Figure S5 **Cytokines WT vs Nod2 KO littermates.** Colon tissue homogenates were prepared and the indicated cytokine concentrations determined by ELISA as outlined in Materials and Methods. *p<0.05, **p<0.01, ***p<0.001: 1 way ANOVA with Bonferroni's multiple comparison test. Means +/− SEM, n = 5–11.
(TIF)

Figure S6 **Serum antibody levels in WT vs Nod2 KO littermates.** Serum was taken from WT and Nod2 KO mice treated or not with DSS in the drinking water. IgA, IgG1, and IgG2a levels in the serum were quantified by ELISA. Bars are mean +/− SEM, n = 7–9. *p≤0.05, 1 way ANOVA with Bonferroni's multiple comparison test.
(TIF)

Figure S7 **Sequence distribution and rarefaction plots for 16S rRNA microbiota analysis.** WT and Nod2 KO littermates were treated with or without DSS in the drinking water as indicated. 16S rRNA libraries were prepared as indicated in Materials and Methods, sequenced and classified using Mothur and a reference Silva alignment. Rarefaction curves were determined using Mothur at the indicated sequence identity cutoffs.
(TIF)

Figure S8 **Classification of 16S rRNA sequences derived from WT and Nod2 KO littermates.** Mice were treated or not with DSS and the colon removed on day 42 post-DSS. The sequences were classified using Mothur using the RDP classification scheme, the confidence values for genus assignment are shown. These are based on the output from Mothur using the latest release of the Silva reference alignment and the RDP classification scheme.
(TIF)

Figure S9 **Phylum and Genus classification of full length 16S rRNA sequences from WT and Nod2 KO mouse colon tissue 42 days post DSS or control as indicated.** Full length 16S rRNA sequence libraries were generated from DNA extracted from colon tissue and analysed in the bioinformatics pipeline as described in Materials and Methods. The sequences were classified using the classifier build within Mothur. Phylum and genus level classifications of these sequences are shown for each group of the mouse model. (Red: NOD2 KO H2O control, Blue: NOD2 KO 42 Days post-DSS, Green: WT H2O control, Yellow: WT 42 Days post-DSS).
(TIF)

Figure S10 **Bioinformatic pipeline used for analysis of 16S rRNA sequences.** See **Materials and Methods** for details.
(TIF)
