# Supplementary figures and images for: Host Genetics and Environmental Factors Regulate Ecological Succession of the Mouse Colon Tissue-Associated Microbiota
Source: PLoS One. 2012 Jan 17;7(1):e30273. doi: 10.1371/journal.pone.0030273 (PMC3260280; doi:10.1371/journal.pone.0030273)

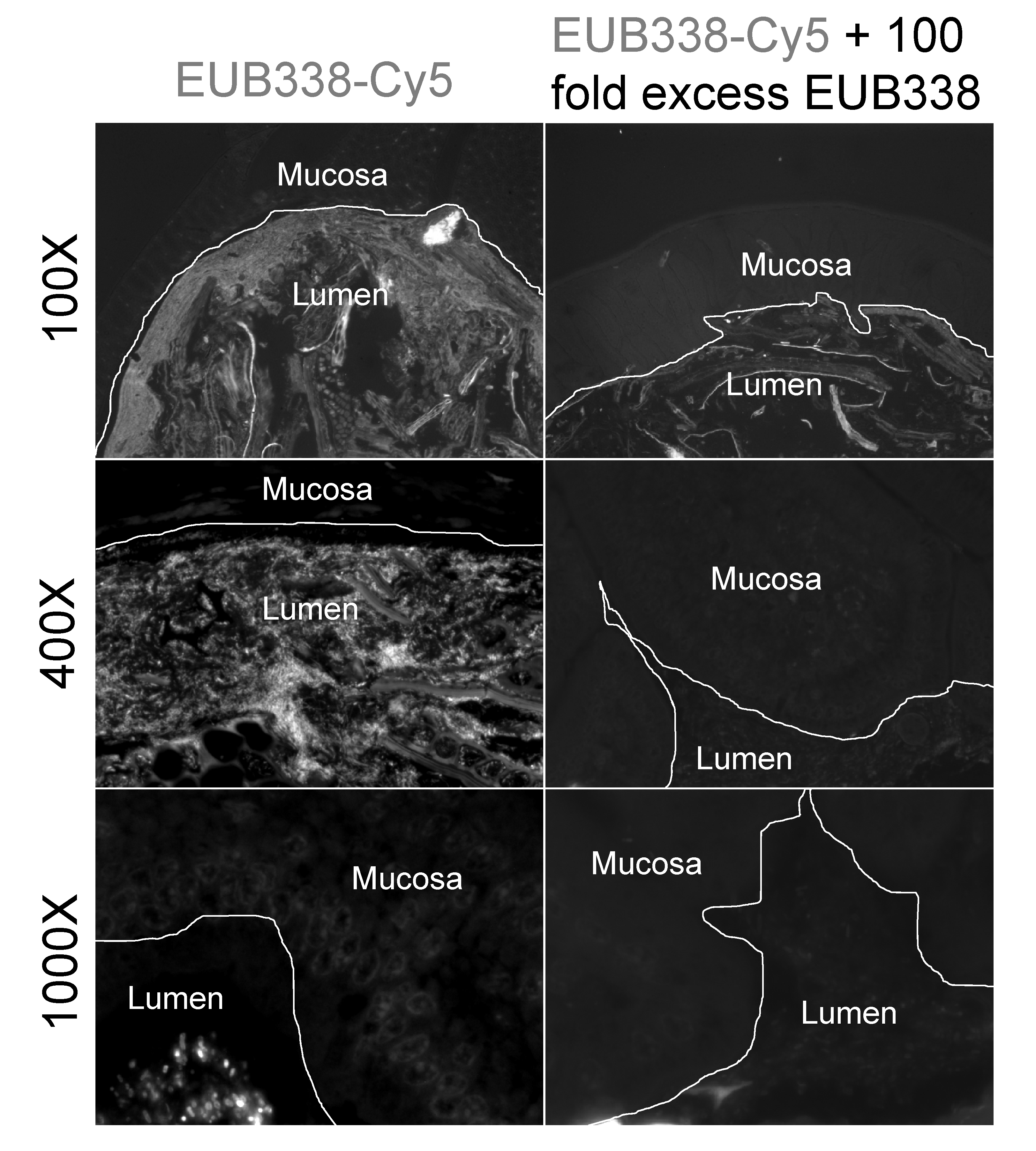

Supplement: Figure S1 — Bacterial FISH probe validation. Formalin-fixed paraffin-embedded colon samples were processed and probed with EUB338 FISH probest for 16S rRNA as described in Experimental Procedures. Left panels: Cy5 probes only. Right panels: Cy5 probes in the presence of 100× excess ‘cold’ EUB338 probes to assess non-specific binding. (TIF) [file pone.0030273.s001.tif]

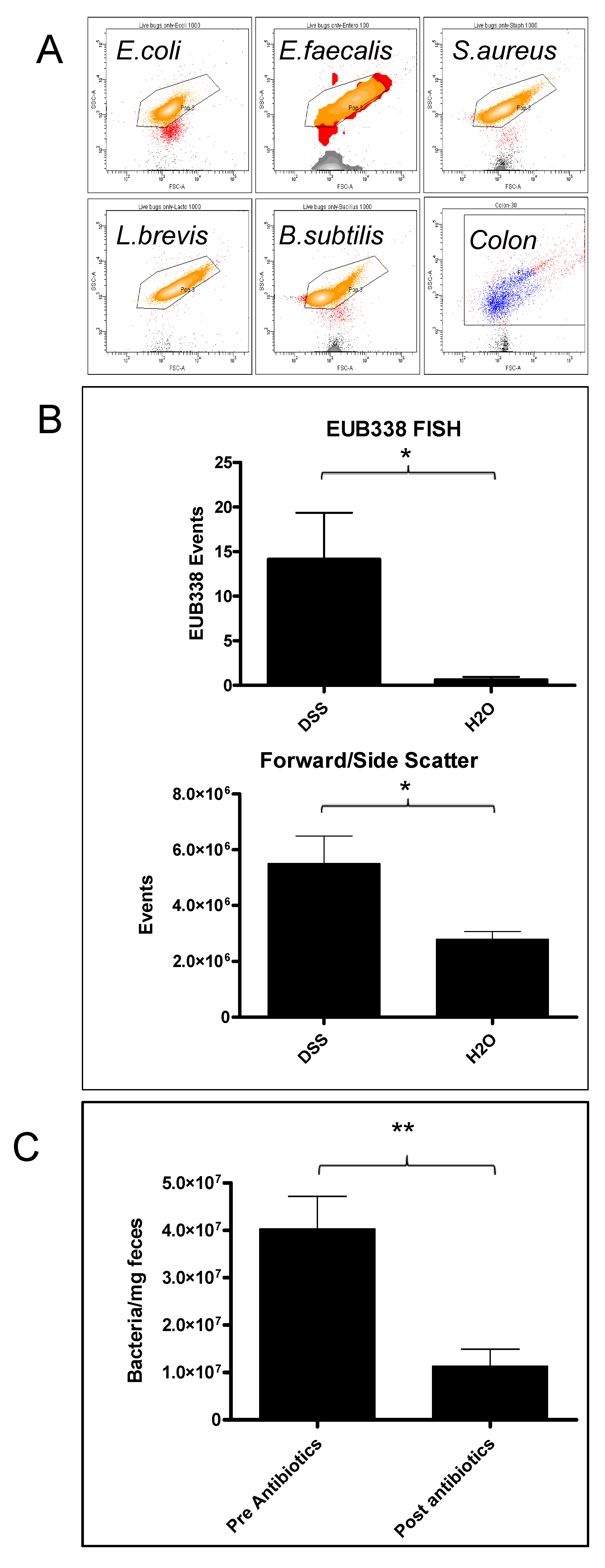

Supplement: Figure S2 — Validation of FACS-based bacterial quantitation. A. Gate selection (forward/side scatter: yellow) for bacterial quantitfication using cultured bacteria and overlap with fecal bacteria (in blue). B: Validation of quantification by comparison with EUB338 FISH probe. Both methods gave comparable statistical significance (*p≤0.05: Students T test) using samples from day 42 post-DSS Nod2 KO mouse colon. C: Validation of FACS bacterial quantification. Fecal matter from mice prior to (Pre-antibiotics) and 3 days following (Post-antibiotics) treatment with broad range antibiotic cocktail was processed and bacteria quantified by the forward/side scatter gate. (**p≤0.01: Students T test). (TIF) [file pone.0030273.s002.tif]

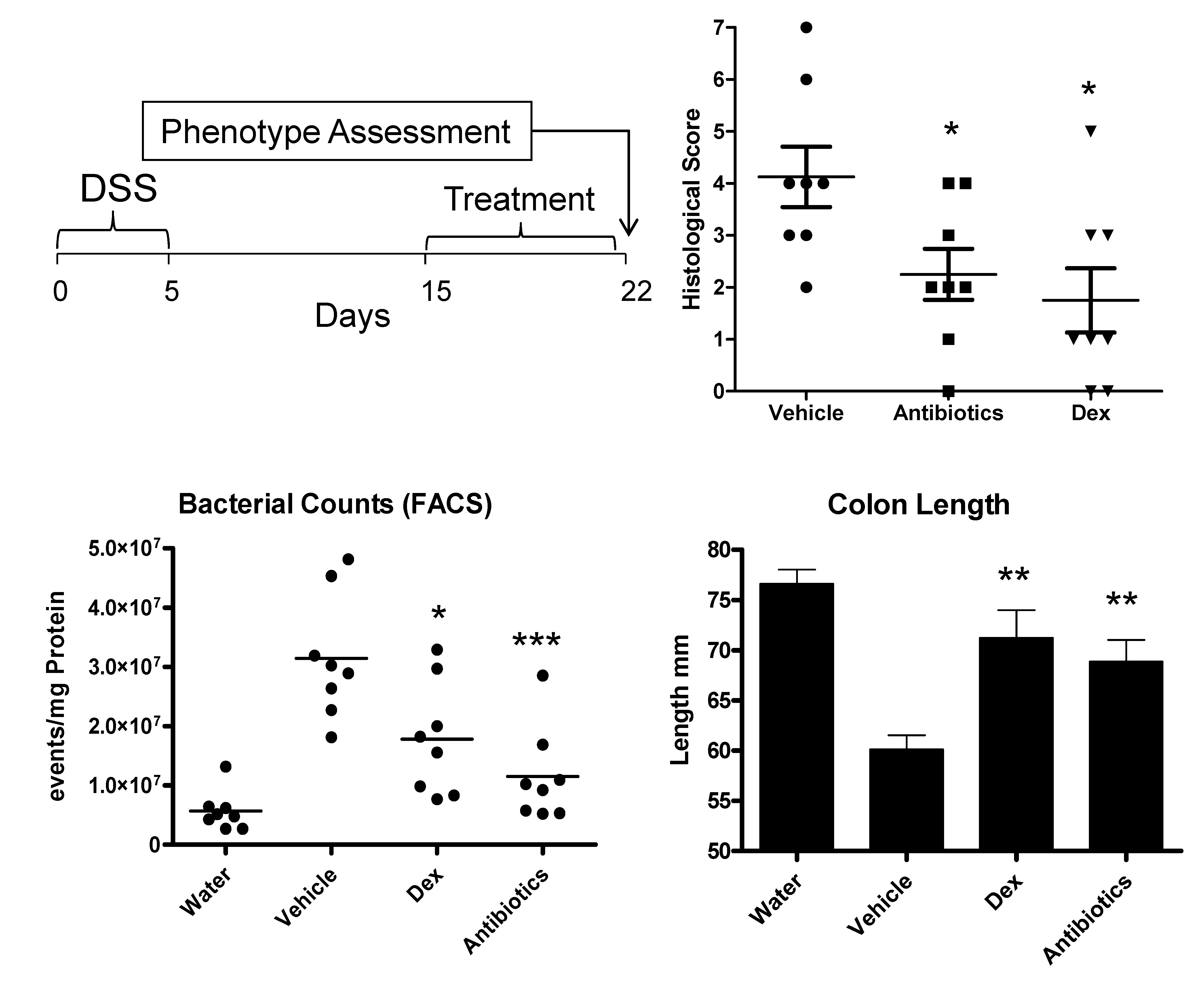

Supplement: Figure S3 — Both antibiotics and steroid treatment significantly impact damage and bacterial tissue penetration. Top left: time line for antibiotic/dexamethasone experiment. Top right: Histological score following therapeutic treatment. *p≤0.05 Students T test vs Vehicle. Bottom left: colon tissue-associated bacterial counts by FACS. *p≤0.05, ***p≤0.001 vs Vehicle group. Bottom right: Colon length (mean +/− SEM; n = 7,8) as physical parameter for tissue damage. **p≤0.01 Students T test vs Vehicle group. (TIF) [file pone.0030273.s003.tif]

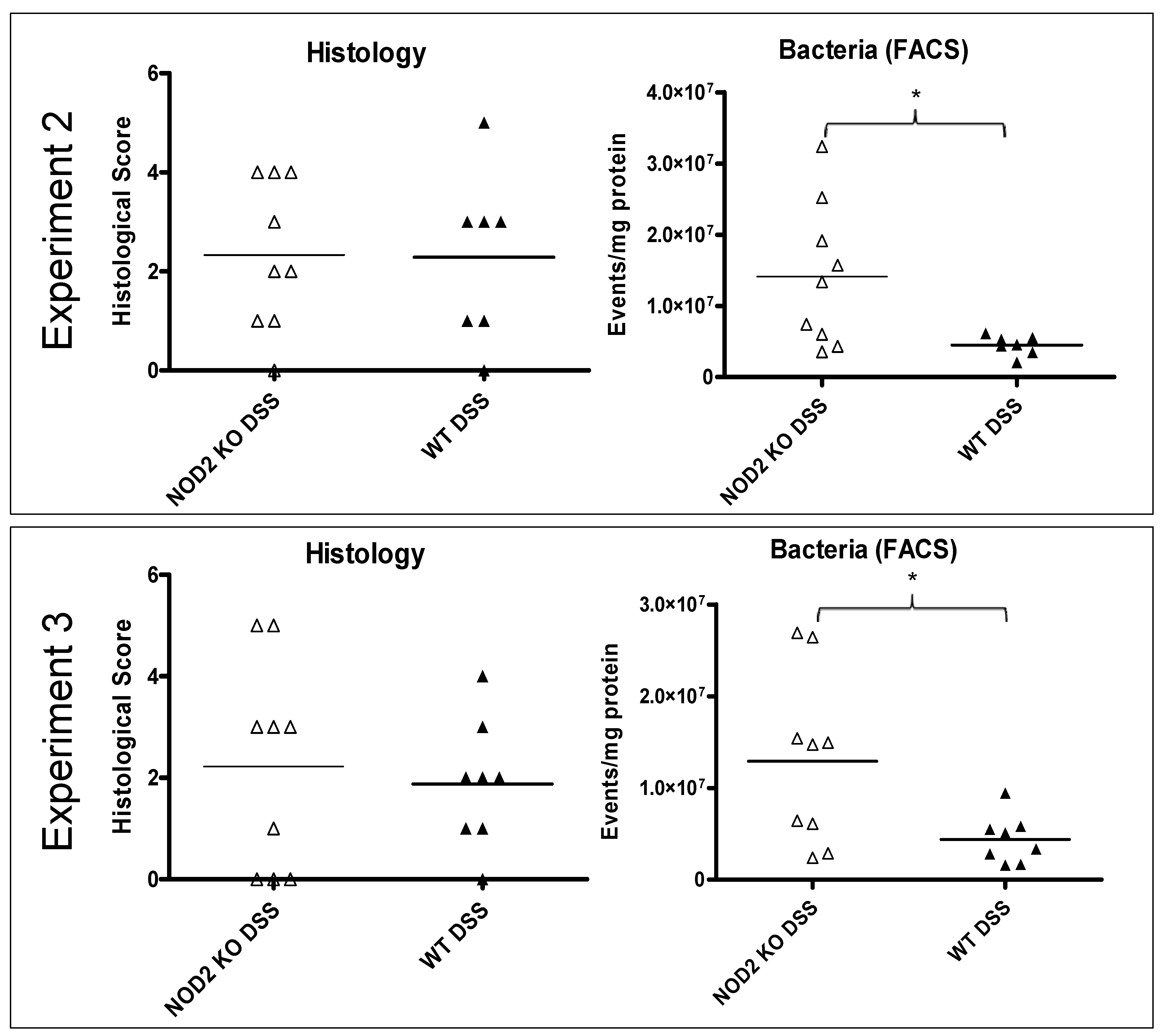

Supplement: Figure S4 — Histology and bacterial load assessment of WT and Nod2 KO littermates following DSS damage. Histology score summary (left) and colon tissue-associated bacterial loads (right) assessed by FACS 42 days following DSS damage. * = p≤0.05 by Students T test. See Figure 4 for additional independent experimental data. (TIF) [file pone.0030273.s004.tif]

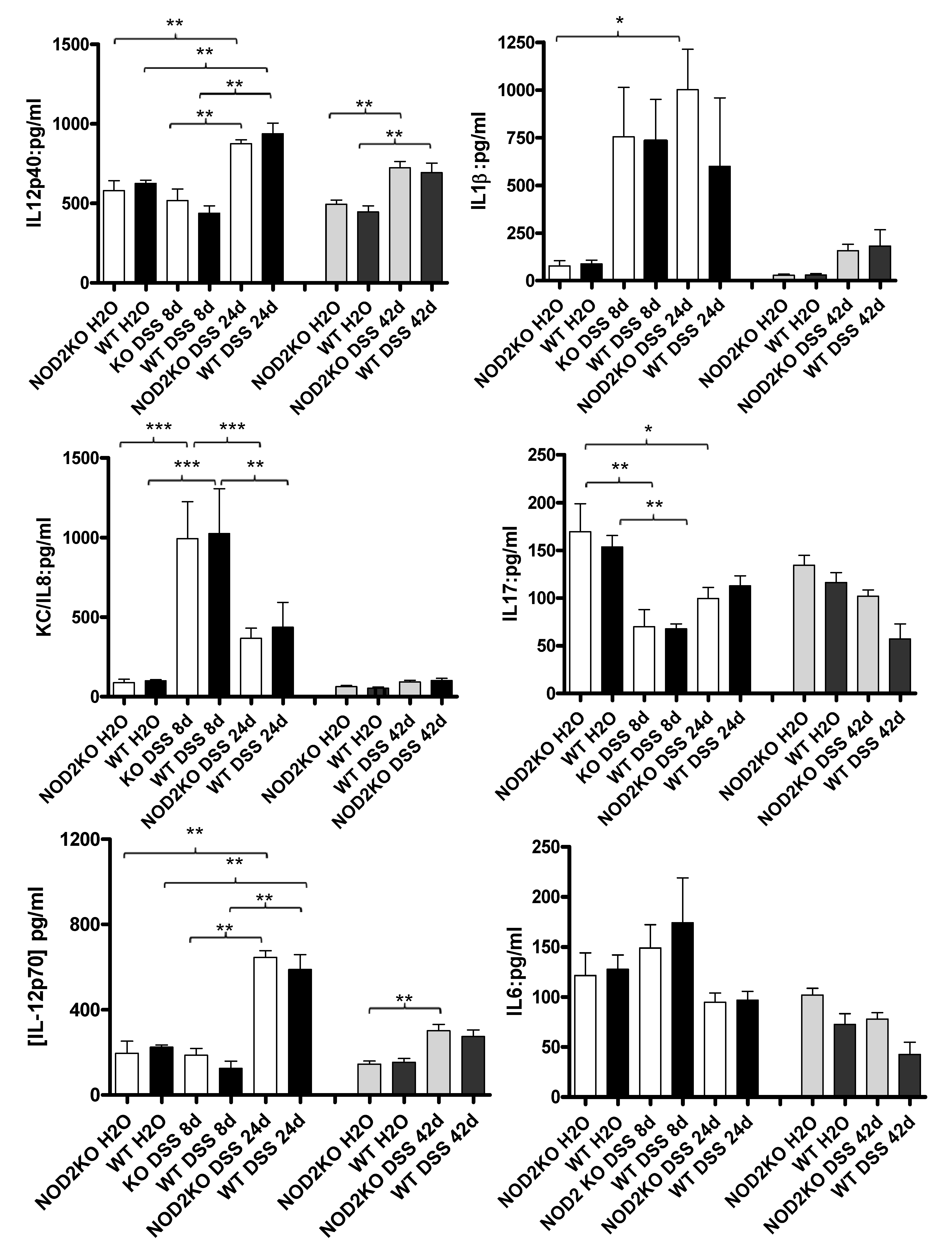

Supplement: Figure S5 — Cytokines WT vs Nod2 KO littermates. Colon tissue homogenates were prepared and the indicated cytokine concentrations determined by ELISA as outlined in Materials and Methods. *p<0.05, **p<0.01, ***p<0.001: 1 way ANOVA with Bonferroni's multiple comparison test. Means +/− SEM, n = 5–11. (TIF) [file pone.0030273.s005.tif]

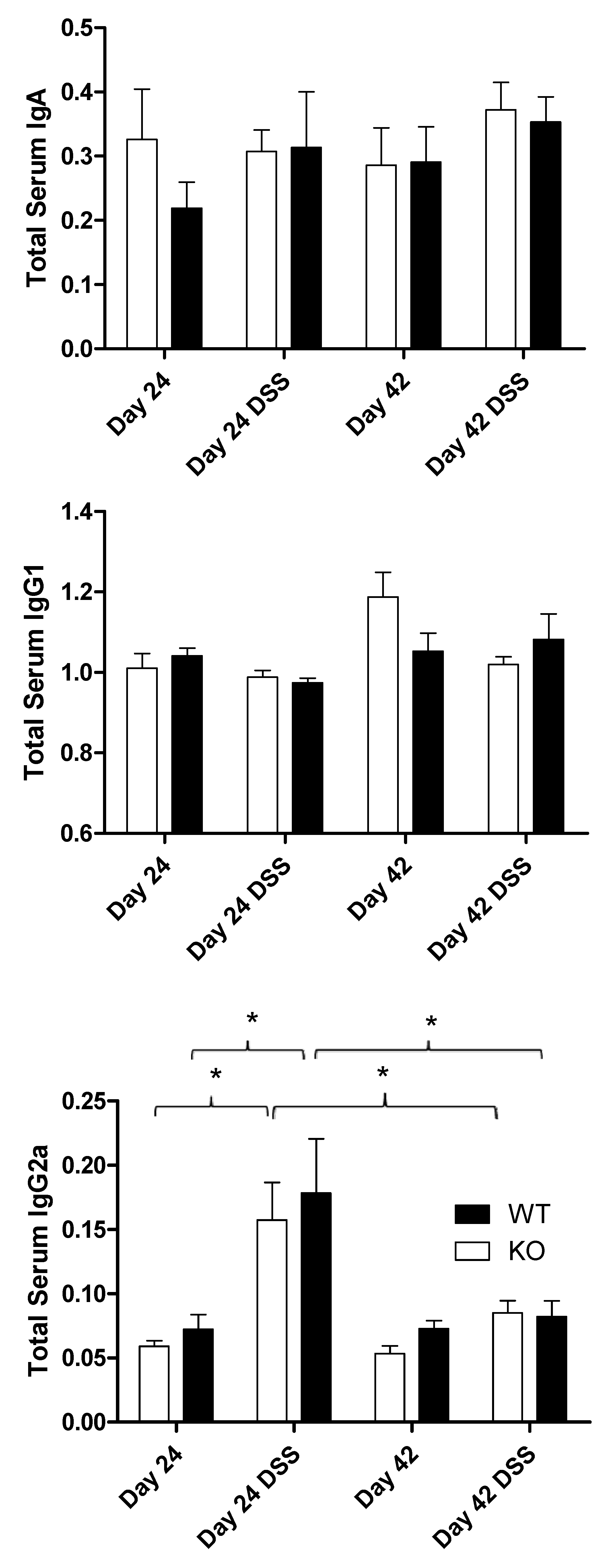

Supplement: Figure S6 — Serum antibody levels in WT vs Nod2 KO littermates. Serum was taken from WT and Nod2 KO mice treated or not with DSS in the drinking water. IgA, IgG1, and IgG2a levels in the serum were quantified by ELISA. Bars are mean +/− SEM, n = 7–9. *p≤0.05, 1 way ANOVA with Bonferroni's multiple comparison test. (TIF) [file pone.0030273.s006.tif]

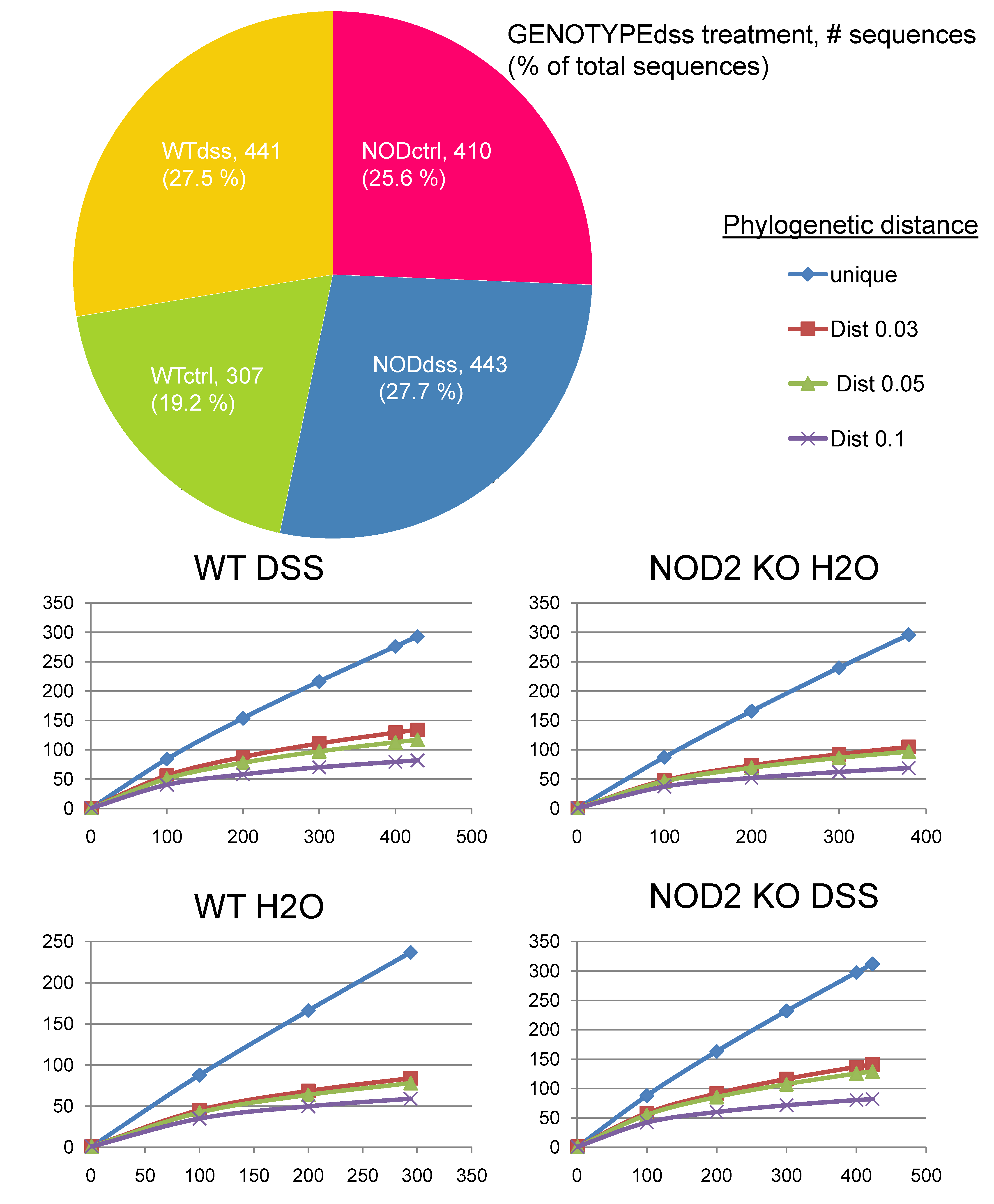

Supplement: Figure S7 — Sequence distribution and rarefaction plots for 16S rRNA microbiota analysis. WT and Nod2 KO littermates were treated with or without DSS in the drinking water as indicated. 16S rRNA libraries were prepared as indicated in Materials and Methods, sequenced and classified using Mothur and a reference Silva alignment. Rarefaction curves were determined using Mothur at the indicated sequence identity cutoffs. (TIF) [file pone.0030273.s007.tif]

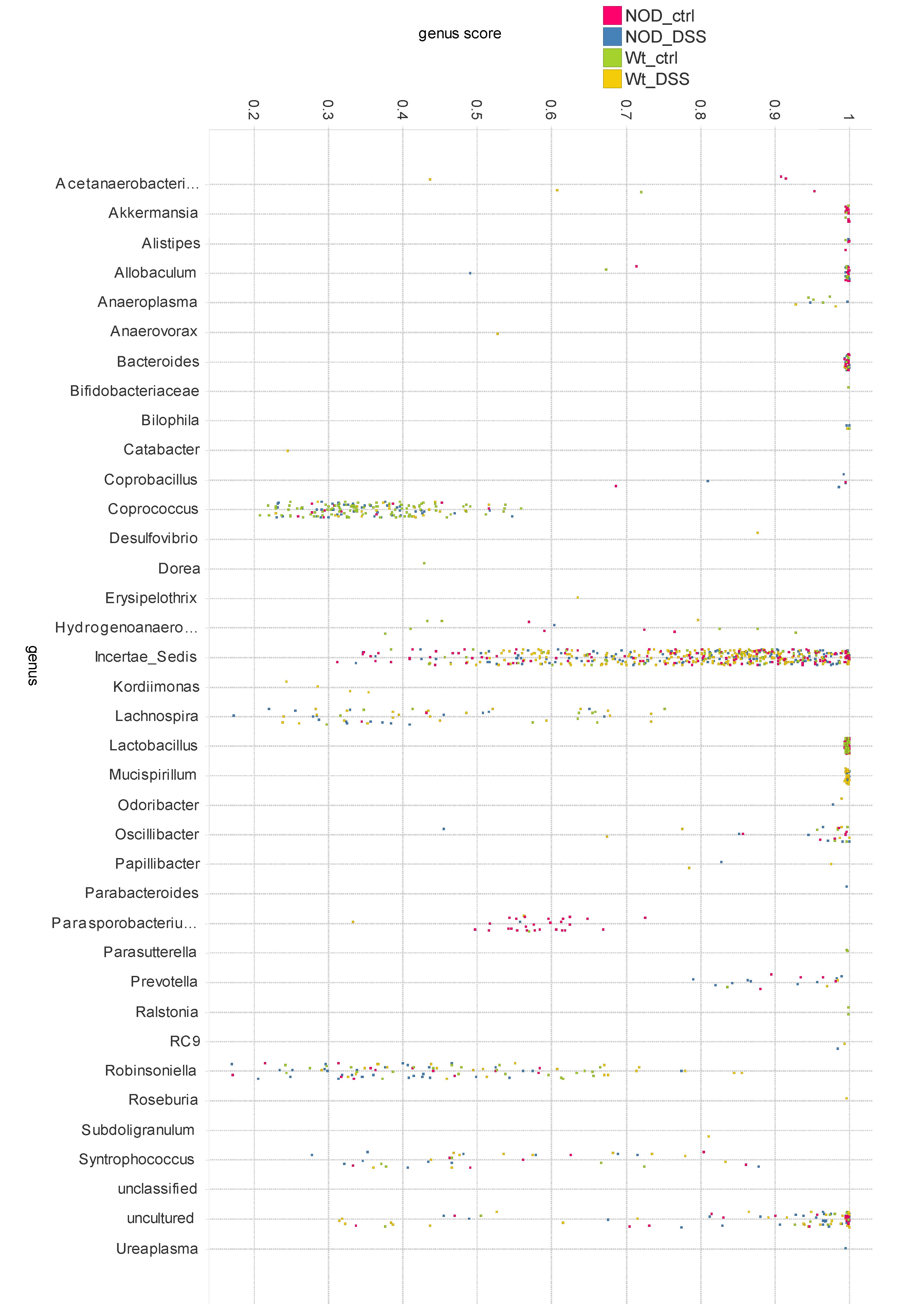

Supplement: Figure S8 — Classification of 16S rRNA sequences derived from WT and Nod2 KO littermates. Mice were treated or not with DSS and the colon removed on day 42 post-DSS. The sequences were classified using Mothur using the RDP classification scheme, the confidence values for genus assignment are shown. These are based on the output from Mothur using the latest release of the Silva reference alignment and the RDP classification scheme. (TIF) [file pone.0030273.s008.tif]

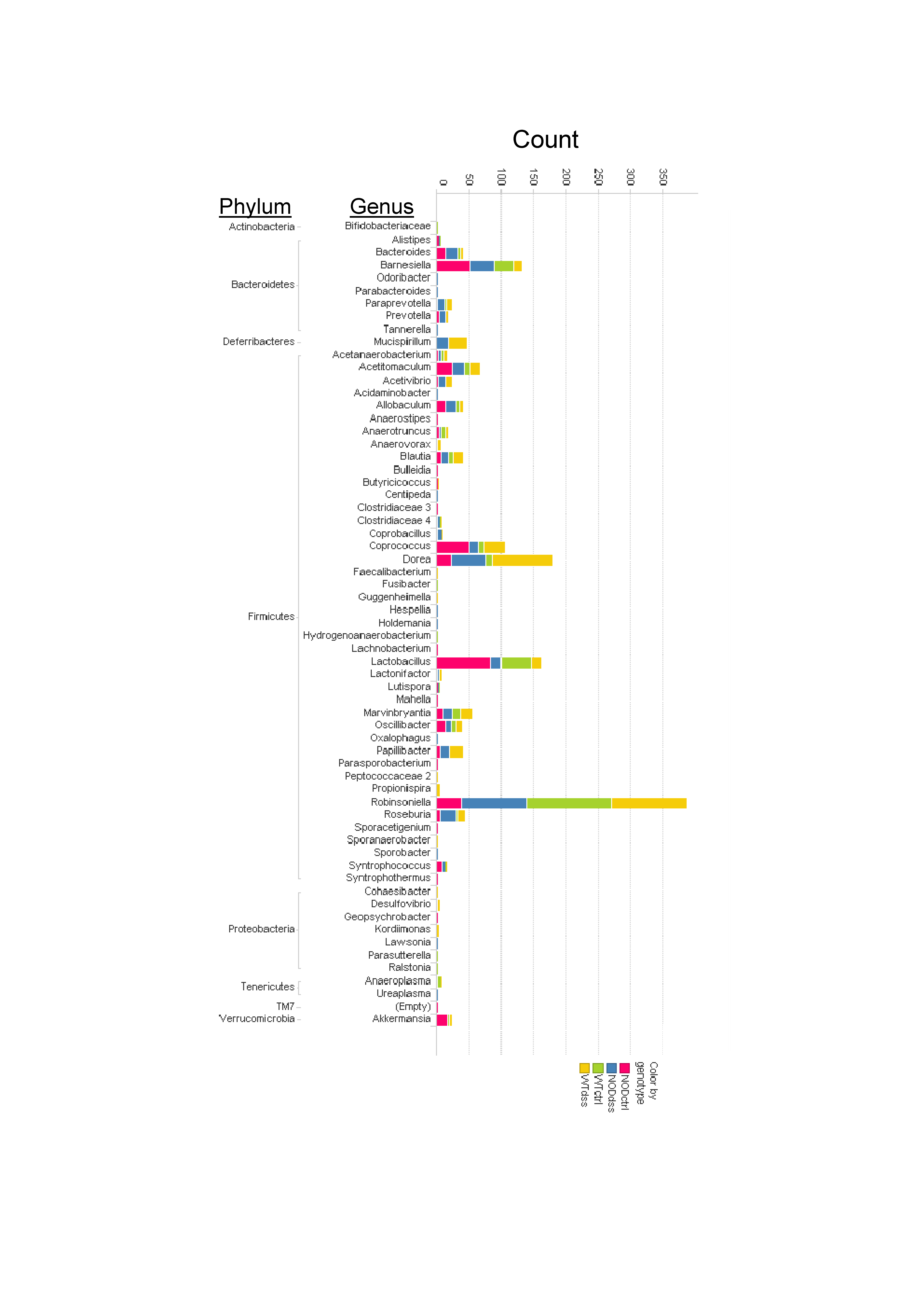

Supplement: Figure S9 — Phylum and Genus classification of full length 16S rRNA sequences from WT and Nod2 KO mouse colon tissue 42 days post DSS or control as indicated. Full length 16S rRNA sequence libraries were generated from DNA extracted from colon tissue and analysed in the bioinformatics pipeline as described in Materials and Methods. The sequences were classified using the classifier build within Mothur. Phylum and genus level classifications of these sequences are shown for each group of the mouse model. (Red: NOD2 KO H2O control, Blue: NOD2 KO 42 Days post-DSS, Green: WT H2O control, Yellow: WT 42 Days post-DSS). (TIF) [file pone.0030273.s009.tif]

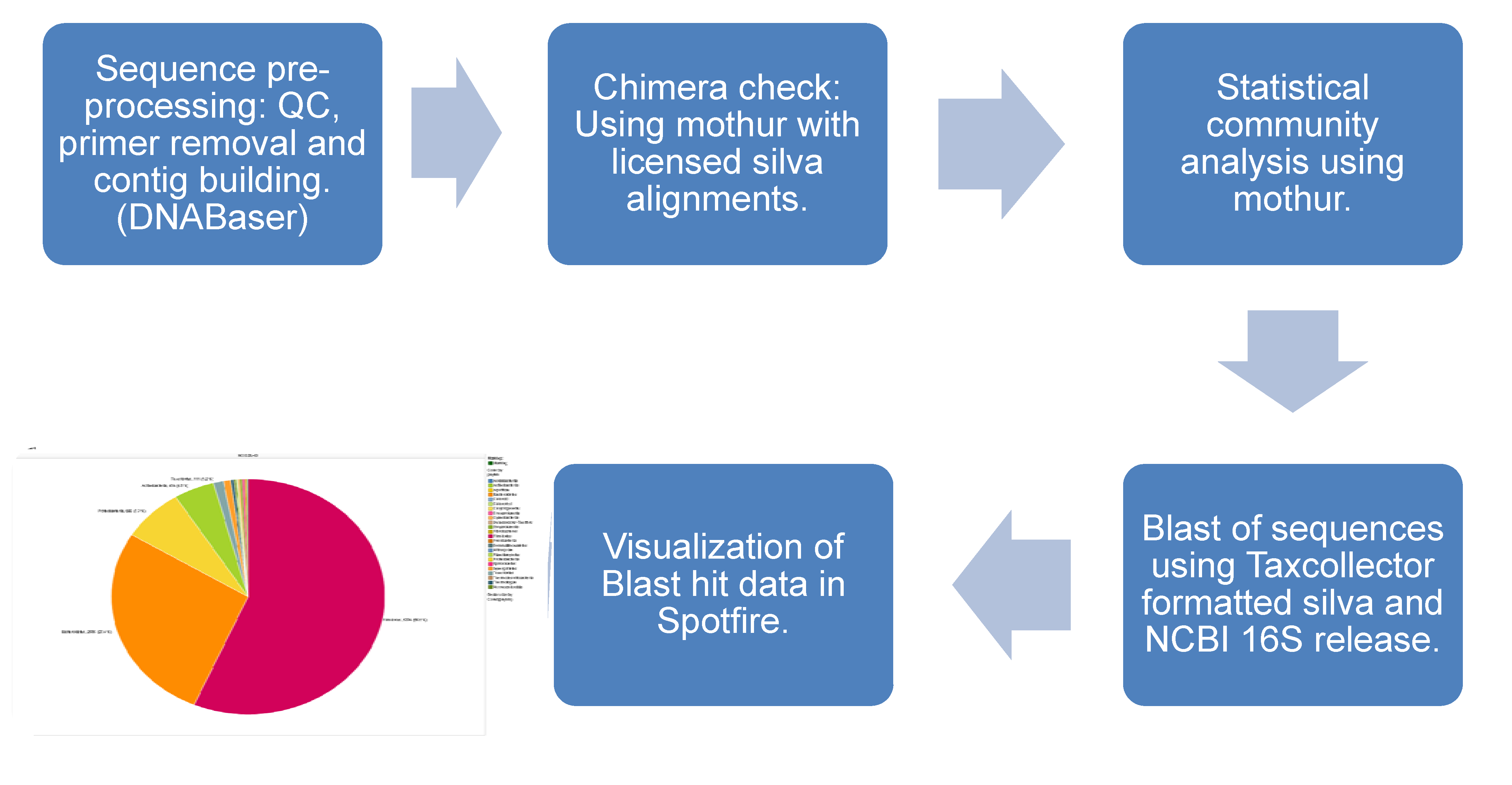

Supplement: Figure S10 — Bioinformatic pipeline used for analysis of 16S rRNA sequences. See Materials and Methods for details. (TIF) [file pone.0030273.s010.tif]
